# Vitamin D status and intake in early school-aged children is tracking from pregnancy—the Swedish GraviD-Child study

**DOI:** 10.1186/s12887-026-06914-3

**Published:** 2026-04-27

**Authors:** Mathilda Forsby, Linnea Bärebring, Anna Amberntsson, Frida Dangardt, Anna Winkvist, Hanna Augustin

**Affiliations:** 1 Department of Internal Medicine and Clinical Nutrition, Institute of Medicine, University of Gothenburg, Gothenburg, Sweden; 2 Department of Molecular and Clinical Medicine, Institute of Medicine, University of Gothenburg, Gothenburg, Sweden; 3 Children’s Heart Center, The Queen Silvia Children’s Hospital, Sahlgrenska University Hospital, Gothenburg, Sweden

**Keywords:** 25-hydroxyvitamin D, Micronutrients, Children, Pregnant women, Observational study, Longitudinal study

## Abstract

**Background:**

The foetus depends on maternal vitamin D status during pregnancy, and the strong correlation between maternal and newborn vitamin D status is well established. However, the long-term tracking of vitamin D status and intake from pregnancy and to childhood remains largely unexplored. We aimed to evaluate vitamin D status and its determinants in early school-aged children, and to explore the tracking of vitamin D status and intake from pregnancy to childhood.

**Methods:**

The Swedish GraviD-Child study includes data of vitamin D status and intake from the mothers first trimester of pregnancy to their children at age 8 years. Concentrations of 25-hydroxyvitamin D (25OHD) were analysed in the first and third trimesters and in children at age 8 years. Vitamin D intake from vitamin D-rich foods was reported in the third trimester, and in children at the ages 5 and 8 years. Candidate determinants of 25OHD concentrations in the children at age 8 years were collected through questionnaires and clinical assessments and analysed using multivariable linear regression. Associations between season-corrected 25OHD concentrations and vitamin D intake during pregnancy and childhood were investigated by Spearman correlations.

**Results:**

Among the 145 children included in the analysis, the mean (SD) 25OHD concentration at age 8 was 67.8 (15.6) nmol/L, with the majority (91.7%) having levels ≥ 50 nmol/L. The only statistically significant determinant of 25OHD concentrations at age 8 were season of blood sampling, with sampling during May to October being associated with higher 25OHD concentrations (*p* < 0.001). Positive correlations were observed between season-corrected 25OHD concentrations in mothers first trimester and in children at age 8 (rho = 0.2, *p* = 0.022). Reported vitamin D intake in the third trimester was positively correlated with intake in children at ages 5 (rho = 0.21, *p* = 0.017) and 8 years (rho = 0.19, *p* = 0.04), with intake at age 5 also positively correlated with intake at age 8 (rho = 0.41, *p* < 0.001).

**Conclusion:**

Among early school-aged children in Sweden with generally adequate vitamin D status, season is an important determinant. In addition, vitamin D status and intake seems to track from pregnancy to childhood.

**Trial registration:**

(NCT05228925|| https://www.clinicaltrials.gov/|| Registration Date 2021–11-18).

**Supplementary Information:**

The online version contains supplementary material available at 10.1186/s12887-026-06914-3.

## Background

Tracking refers to the stability of a measurement over time, reflecting whether relative ranks within the population are maintained. There is a growing body of evidence suggesting that maternal vitamin D status during pregnancy closely tracks with vitamin D status in the newborn at birth and in the early postnatal period [1–3]. Moreover, vitamin D status at birth has been shown to track into later life, as demonstrated by a Danish mother–child cohort in which cord blood vitamin D concentrations were associated with levels in children at 4 years of age [4]. Nevertheless, research investigating the long-term tracking of vitamin D status and intake from pregnancy to childhood is yet to be evaluated. If vitamin D status tracks from pregnancy to childhood, the prenatal period may offer an early opportunity for targeted vitamin D interventions among those at risk. Preventing vitamin D insufficiency or deficiency may have potential implications for long-term vitamin D status, i.e., through sustained dietary and supplementation patterns. Vitamin D deficiency in children can, over time, lead to impaired bone mineralisation, and in its most severe form, rickets [5]. Given the importance of vitamin D in bone health, it has been suggested that prenatal vitamin D exposure may influence the skeletal development of the child [6]. Indeed, results from intervention studies indicate that vitamin D supplementation during pregnancy have a beneficial effect on bone mineral density in early childhood [7], with a persistent effect up to the age of 6–7 years [8, 9].

Vitamin D status is typically assessed using the biomarker 25-hydroxyvitamin D (25OHD), due to its relatively long circulating half-life and its capacity to reflect both cutaneous synthesis and intake from food and supplements [10]. Variations in 25OHD concentrations can be attributed to seasonal and geographical factors [11]. At higher latitudes, such as the Nordic countries, the insufficient UVB radiation for dermal synthesis of vitamin D during the winter months necessitates the intake of vitamin D from dietary sources and supplements [11, 12]. Additional factors influencing cutaneous vitamin D synthesis and 25OHD concentrations includes skin pigmentation [13, 14], time spent outdoors [15–17], and travel to sunny countries [17, 18]. Additionally, vitamin D supplementation [17, 19–22], vitamin D intake from food [13, 14, 19, 23–25], socioeconomic status [15, 16, 22, 26], age [15, 16, 20–22], pubertal stage [15], and body mass index (BMI) [13, 18, 19, 22, 27] may be important determinants for vitamin D status in children.

The number of natural food sources with a high vitamin D content is limited [12]. Consequently, vitamin D fortification is either mandatory or voluntary in some countries [28–30]. In Sweden, the mandatory fortification policy was updated in 2018 to include fortification of vitamin D in margarine, milk with ≤ 3% fat by weight, and fermented milk products (e.g., yoghurt and sour milk) with ≤ 3% fat by weight [31, 32]. Moreover, a daily vitamin D supplement of 10 µg is recommended for children up to the age of two [33]. This recommendation extends to children with low vitamin D intake from food sources or sun exposure, for whom vitamin D supplementation should continue beyond the age of two [33]. Vitamin D intake and status among school-aged children in Sweden were reported in the most recent national dietary survey in 2016–17, which found that only 16% of the children reported vitamin D intakes above the average requirement (7.5 µg/day), and 42% had 25OHD concentrations < 50 nmol/l [19]—a threshold commonly used to indicate vitamin D insufficiency [12, 34]. However, vitamin D intake and status in children in this age group have not been assessed since the expansion of the Swedish vitamin D fortification programme in 2018.

The aim of this study was to evaluate vitamin D status and its determinants in early school-aged children, and to explore the tracking of vitamin D status and intake from pregnancy to childhood.

## Methods

### Study design and recruitment

The GraviD study is a mother–child cohort conducted in south-west Sweden, following pregnant women and their children until the age of 8 years [35, 36]. Pregnant women who had not exceeded 16 gestational weeks and were attending routine antenatal care in Gothenburg, Södra Älvsborg, or Södra Bohuslän were invited to participate in the study. The recruitment was conducted in two periods, in fall 2013 and in spring 2014, resulting in a final sample of 2125 pregnant women.

**Fig. 1:**
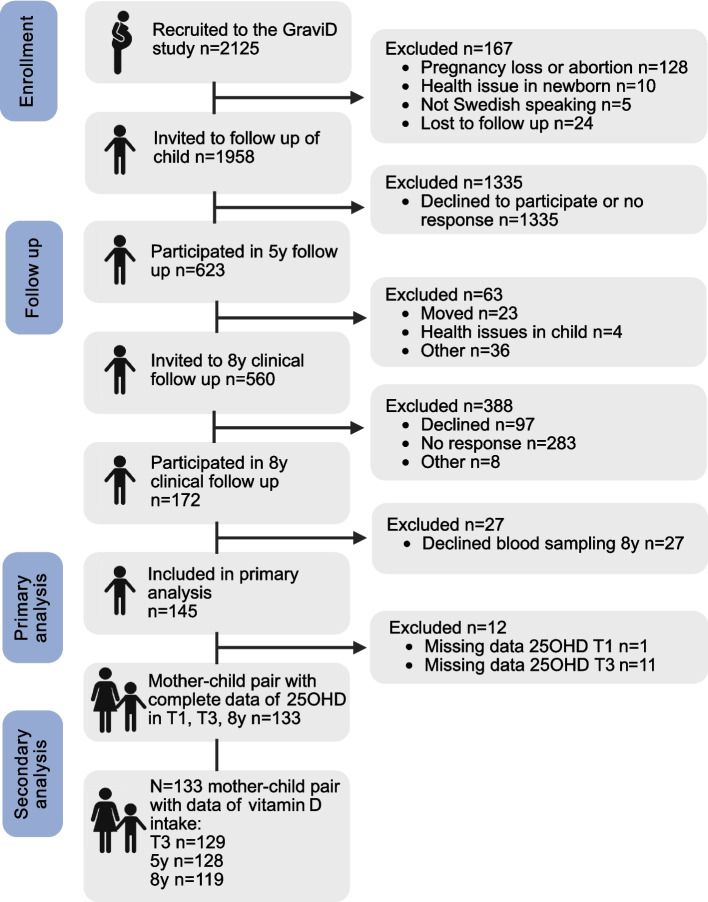
Flowchart illustrating the recruitment and selection of the mother–child pairs included in the GraviD-Child study and the subsequent analyses. Created with BioRender.com. Abbreviations: y, years; 25OHD, 25-hydroxyvitamin D; T1, first trimester; T3, third trimester

When the children born in the GraviD study reached 5 years of age, the families were invited (*n* = 1958) to participate in a follow up using the child’s medical records and a questionnaire on health and dietary habits at age 5 years. The invitation was sent by post and written in Swedish. Families who participated in the 5-year follow up were subsequently invited to a clinical follow up of the child’s health at age 8 years. The invitation was sent by post and written in Swedish. Those who had consented to participate were also invited to a clinical follow up of the child’s health at age 8 years. The clinical follow up were conducted at Queen Silvia Children’s Hospital in Gothenburg, Sweden, between 2022–2023. A total of 172 children participated in the clinical follow up. The recruitment and selection of the included mother–child pairs are illustrated in Fig. 1.

The study information was provided in both verbal and written form. Written study information adapted to children was also provided. Written informed consent was obtained from both parents, and the participants were informed of the option of withdrawing their consent or participating in only some of the examinations. The procedures conducted in the GraviD study were performed in accordance with the Declaration of Helsinki and had ethical approval from the Regional Ethics Committee in Gothenburg and the Swedish Ethical Review Authority (897‐11, T439‐13, T085‐14, 2019‐05219, 2021–04194, 2022–01361-02). The GraviD-Child clinical follow up is registered at ClinicalTrials.gov under the identification number NCT05228925 (Registration Date: 2021–11–18). The study adhered to the Strengthening the Reporting of Observational Studies in Epidemiology (STROBE) guidelines for reporting observational studies.

### Data collection during pregnancy

Maternal data were collected at a routine antenatal care visit in first (gestational week < 17) and third trimester (gestational week > 31). Participants answered questionnaires including information on their country of origin, education level, sun exposure, and intake of vitamin D-rich foods and supplements. Vitamin D intake from vitamin D-rich foods in third trimester included oily fish, margarine, milk, and fermented milk (e.g., yoghurt and sour milk). Information about the questionnaire of vitamin D-rich foods has been described in detail elsewhere [37].

Data on maternal age, tobacco use, height, weight, parity, and child’s sex were retrieved from antenatal care and obstetrics medical journals. Non-fasting venous blood sample was taken in both first and third trimester. Serum 25OHD concentrations were determined by liquid chromatography-tandem mass spectrometry (LC–MS/MS) at the clinical laboratory in Malmö, Sweden. The procedures for blood sample handling in the GraviD study and the analytical performance of the LC–MS/MS assay have been described previously in detail [35]. The assay was calibrated using a commercial calibration standard (Chromsystems) traceable to the National Institute of Science and Technology (NIST) standard reference material (SRM) 972. The method demonstrated linearity across the measuring range (25OHD3 6–450 nmol/L; 25OHD2 6–225 nmol/L). The inter-assay coefficient of variation was 6% at 40 nmol/L. The laboratory met the performance targets set by the Vitamin D External Quality Assessment Scheme (DEQAS) [38] during the analysis period. Assay bias relative to the DEQAS reference measurement procedure target values ranged from −0.1 to + 13.8%.

### Data collection of the children in the GraviD-Child follow up

The GraviD-Child follow up was conducted when the children were 5 and 8 years old. Data collected at age 5 included health and growth information from medical records and a health- and food habits questionnaire. At age 8, the children took part in a clinical follow up involving estimation of dietary intake and measurements of body composition, nutritional status, and vascular health [36]. The clinical follow up was conducted at one study visit. All examinations were performed by trained study personnel and in company with a parent.

### Questionnaire data at 8 years of age

A questionnaire was sent to the families before the clinical follow up at 8 years of age, where the parents were instructed to complete the questions in advance and bring it to the study centre. The questionnaire included information of children’s health, eye colour, skin pigmentation, eating habits, and sun exposure. Questions about sun exposure included the number of hours spent outdoors outside school in summer and winter, and any travel to < 40° North in the previous six months. Skin pigmentation was assessed using a question regarding the child’s reaction to the first hours of sun exposure in spring. The response options were based on the Fitzpatrick’s scale [39] and included: always burns, never tans; always burns, tans with difficulty; sometimes mild burn, tans about average; rarely burns, tans easily; minimally burns, tans very easily; and never burns, tans very easily.

The questionnaire included questions about the children’s use of supplements, including frequency (6–7 times per week; 4–5 times per week; 1–3 times per week; < 1 time per week; never) and dosage of fish liver oil, multivitamin, and omega-3 supplements (1 dose per occasion; 2 doses per occasion; ≥ 3 doses per occasion). If other supplements were used, participants were asked to provide the type, dosage and frequency of use. The type of fish liver oil, multivitamin, and omega-3 supplements was not specified. The child was considered as a vitamin D supplement user if any use of vitamin D supplements (including use of multivitamins) was reported, regardless of frequency and dosage.

### Intake of vitamin D-rich foods at 5 and 8 years of age

Vitamin D intake from vitamin D-rich foods was estimated using a short vitamin D questionnaire at the ages of 5 and 8 years. Participants were asked to report their intake of oily fish, margarine spreads, milk, and fermented milk (e.g., sour milk and yoghurt), during the previous two months. A total of 3–5 frequency categories of intakes were provided depending on the food item. Intake of vitamin D-rich foods were converted into vitamin D intake using the national database on nutrient content (version 2024–05–29) from the Swedish National Food Agency [40]. A single serving of oily fish was defined as 50 g, with an estimated vitamin D content of 7.49 µg per 100 g (equivalent to 100 g of salmon). One serving of milk and fermented milk was assumed to be 500, 200, or 50 g, depending on the answer, and assumed to contain 1 µg vitamin D per 100 g. One serving of margarine as a spread was assumed to be 8 g and to contain 20 µg vitamin D per 100 g. The maximum vitamin D intake from vitamin D-rich foods, calculated using these assumptions, was 18.5 µg per day. Vitamin D intake from the short vitamin D questionnaire has been validated in the GraviD dataset using maternal four-day food records and 25OHD levels as reference methods, demonstrating correlations of rho = 0.51 (including all seasons) and rho = 0.137–0.175 (including all seasons and November to April), respectively [41]. However, it has not been validated among children.

### Anthropometry and blood sampling at 8 years of age

Clinical examinations were performed non-fasting [36]. Weight and height were measured three times and averaged. BMI was calculated as weight in kilograms divided by the square of height in metres. BMI z-scores and weight status categories were determined using age- and sex-specific reference data from the International Obesity Task Force [42]. Weight status was categorized as normal weight (including thinness) or overweight (including obesity). Non-fasting venous blood samples were collected (*n* = 145). The samples were centrifuged at 2800 × g for 7 min at room temperature and sent the same day to the Clinical Chemistry Östra, Sahlgrenska University Hospital, Gothenburg, for analysis of 25OHD. Plasma 25OHD concentrations were determined by chemiluminescent microparticle immunoassay (CMIA), using Abbott’s Alinity i, according to the clinical routine. The measurement range of the assay is 8.8–385.5 nmol/L, and the calibrator is traceable to NIST SRM 2972. The coefficient of variation was 8% at 25 nmol/L and 6% at 45 nmol/L. At the time of the analyses (2022–2023) the laboratory participated in the external quality control programme Bio-Rad External Quality Assessment Services (EQAS), but not DEQAS.

### Statistical analysis

Normally distributed data are presented as mean and standard deviation (SD), whereas non-normally distributed data are presented as median and 25th and 75th percentiles. Normality was assessed visually using boxplots and histograms. Categorical data are presented as percentages.

The seasonal variation of crude plasma 25OHD concentrations in children were visualized by a scatterplot with loess smoothing curve and 95% confidence interval. 25OHD concentrations were categorized into < 30 nmol/L, < 50 nmol/L, and ≥ 50 nmol/L. Maternal country of origin was categorized into birth regions: Northern Europe (Nordic countries, Latvia, Lithuania, and the United Kingdom), Continental Europe (rest of Europe), South and North America, Asia, and Africa.

The association between crude plasma 25OHD concentrations in children at age 8 years and potential determinants was visualized in a directed acyclic graph (figure S1). The potential determinants were selected based on previous literature on vitamin D status in children [13–27] and included season of blood sampling (November to April; May to October) vitamin D intake from vitamin D-rich foods (µg per day), use of vitamin D supplements (vitamin D supplement user; non-vitamin D supplement user), time spent outdoors during summer (hours per day), child’s BMI z-score, maternal origin (born in Northern Europe; not born in Northern Europe), and maternal education (university education; no university education). Recent travel to < 40° North and skin pigmentation were also considered as potential determinants but were not included in the model. Travel to < 40° North was excluded in the analysis due to the low number of cases (3.6%). Skin pigmentation was excluded because the data was highly clustered in the middle of the scale, regardless of maternal origin. Determinants of crude 25OHD concentration in children were predicted using multivariable linear regression analysis. The assumption of linearity was visually assessed using scatterplots of 25OHD concentrations in children and the explanatory variables. The assumption of homoscedasticity was visually assessed using a scatterplot of the residuals and fitted values. The normality of the residuals was visually assessed by quantile–quantile plots. The explanatory variables were tested for multicollinearity using a Spearman correlation matrix, where the highest correlation coefficient was 0.11; therefore, we were not concerned about multicollinearity. Our analysis dataset included those that had complete measurements of 25OHD in children at 8 years of age (*n* = 145) (Fig. 1). Missing data on covariates (vitamin D intake from vitamin D-rich foods, *n* = 14 (9.7%); use of vitamin D supplements, *n* = 3 (2.1%); time spent outdoors during summer, *n* = 3 (2.1%)) was handled by multiple imputation with chained equations (MICE) [43], using predictive mean matching for continuous variables and logistic regression for binary variables. Ten imputed datasets were generated.

Spearman correlations with pairwise complete observations were used to assess the tracking of vitamin D status and intake from pregnancy to childhood. As a rank-based analysis, this approach is less sensitive to systematic differences in absolute concentrations between methods. For all analyses of tracking between pregnancy and childhood, 25OHD was season-corrected to account for differences in the seasonality of blood sampling. Season correction was applied by the following procedure:

At each of the three time points (first and third trimesters of pregnancy in mothers and at 8 years of age in children), mean 25OHD concentrations were firstly calculated for all included participants. Secondly, a mean 25OHD concentration was calculated for each calendar month. Finally, the individual 25OHD concentration value was season-corrected based on the percentage difference between the calendar month mean and the mean of all participants over the year. Figure S2 presents scatterplots illustrating the seasonal variation in 25OHD concentrations during the first and third trimesters of pregnancy, as well as in children at 8 years of age. The plots include loess smoothed curves for both the crude 25OHD concentrations and the season-corrected concentrations, along with 95% confidence intervals.

*P*-values below 0.05 were considered statistically significant in all analyses. All analyses were performed in R version 4.3.2 (R Foundation for Statistical Computing, Vienna, Austria).

## Results

### Study population

Characteristics of the *n* = 145 mother–child pairs in the GraviD-Child study are presented in Table 1. Most mothers were born in Northern Europe (86.9%) and had a university education (80.7%). Around half of the children were male (55.9%). Mean (SD) BMI z-score was 0.13 (0.84). Overweight or obesity was observed in 9.9% (*n* = 8) of the males and 7.8% (*n* = 5) of the females at the follow up at age 8 years.

**Table 1:** Characteristics of the *n* = 145 mother–child pairs in the GraviD-Child study

	**Mean (SD) or % (n)**
Mothers	
Age T1, years	33.0 (4.4)
Country of origin, %	
Northern Europe	86.9 (126)
South and North America	2.8 (4)
Continental Europe	4.8 (7)
Asia	4.8 (7)
Africa	0.7 (1)
University education level at T1, %	80.7 (117)
Body mass index at T1, kg/m^2^	23.6 (3.1)
Children at the age of 8 years	
Sex, %	
Female	44.1 (64)
Male	55.9 (81)
Age, years	8.2 (0.2)
Body mass index, kg/m^2^	16.0 (1.74)
Eye colour, %	
Blue/green	73.4 (102)
Hazelnut brown	17.3 (24)
Dark brown	9.4 (13)
Vitamin D supplement user (any), %	28.9 (41)
Recently travelled < 40° North, %	3.5 (5)

*Abbreviations 25OHD* 25-hydroxyvitamin D, *T1* Trimester 1

### Vitamin D status and its determinants in children at age 8 years

**Fig. 2:**
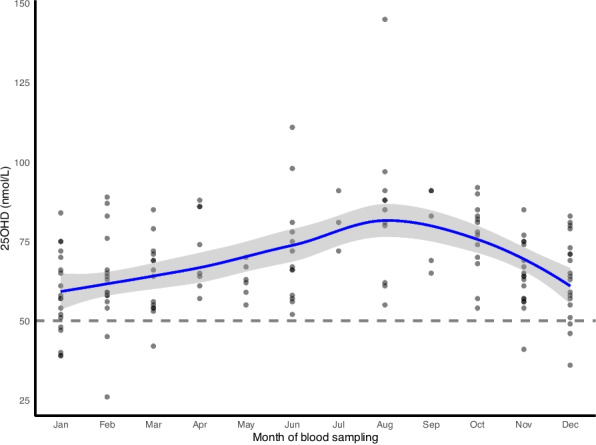
Scatterplot with loess smoothing curve (solid, blue line) and 95% confidence interval (grey area) of the seasonal variation of crude plasma 25-hydroxyvitamin D (25OHD) concentrations in children at 8 years of age (*n* = 145). The dotted, grey line shows the cut-off for 25OHD ≥ 50 nmol/L

Mean (SD) crude 25OHD concentration in children at 8 years of age was 67.8 (15.6) nmol/L. More than half of the blood samples (64.8%, *n* = 94) were drawn during the winter months (November to April). Less than one in ten (8.3%, *n* = 12) had 25OHD concentrations below 50 nmol/L and their blood samples were all drawn in the winter season (Fig. 2). Concentration of 25OHD below 30 nmol/L was observed in only one child.

Median (p25, p75) time spent outdoors during summer was 3 (2, 4) hours per day. About a third reported taking supplements containing vitamin D, commonly a multivitamin-mineral supplement 1–3 days per week.

The determinants of crude 25OHD concentrations in children at mean 8.2 (0.2) years explained 17.4% of the variation in 25OHD concentrations. Only season was statistically significant, with sampling during May to October being associated with higher 25OHD concentrations (*p* < 0.001) (Table 2).

**Table 2:** Multivariable linear regression analysis of determinants of crude 25-hydroxyvitamin D concentrations (nmol/L) in children at 8 years of age (*n* = 145)

**Children at the age of 8 years**	Beta	95% CI
Season of blood sampling		
November to April	Reference	
May to October	12.73	7.64, 17.82
Vitamin D intake from vitamin D-rich foods (µg/day)	0.09	−0.74, 0.92
Vitamin D supplement use		
No	Reference	
Yes	−1.87	−7.40, 3.67
Time spent outdoors during summer (hours/day)	0.39	−0.94, 1.72
Body mass index z-score	−0.35	−3.38, 2.68
Mothers		
Origin		
Born in Northern Europe	Reference	
Not born in Northern Europe	−2.88	−10.40, 4.63
Education level		
University	Reference	
No university education	4.40	−1.82, 10.62

*Abbreviation*: *CI* Confidence interval

### Tracking of vitamin D status and intake from mother’s pregnancy to childhood

Median (p25, p75) season-corrected serum 25OHD concentrations were 71.8 (60.2, 82.6) and 77.7 (62.5, 96.6) nmol/L in the mothers’ first and third trimesters, respectively. Median season-corrected plasma 25OHD concentration was 66.9 (58.9, 75.9) nmol/L in children at 8 year-follow up. The median reported vitamin D intakes from vitamin D-rich foods were 7.1 µg/day (5.3, 9.8) during the mothers’ third trimester, and for the children 5.6 µg/day (3.9, 7.8) at the 5 years of age, and 5.8 µg/day (4.2, 8.1) at 8 years of age.

**Fig. 3:**
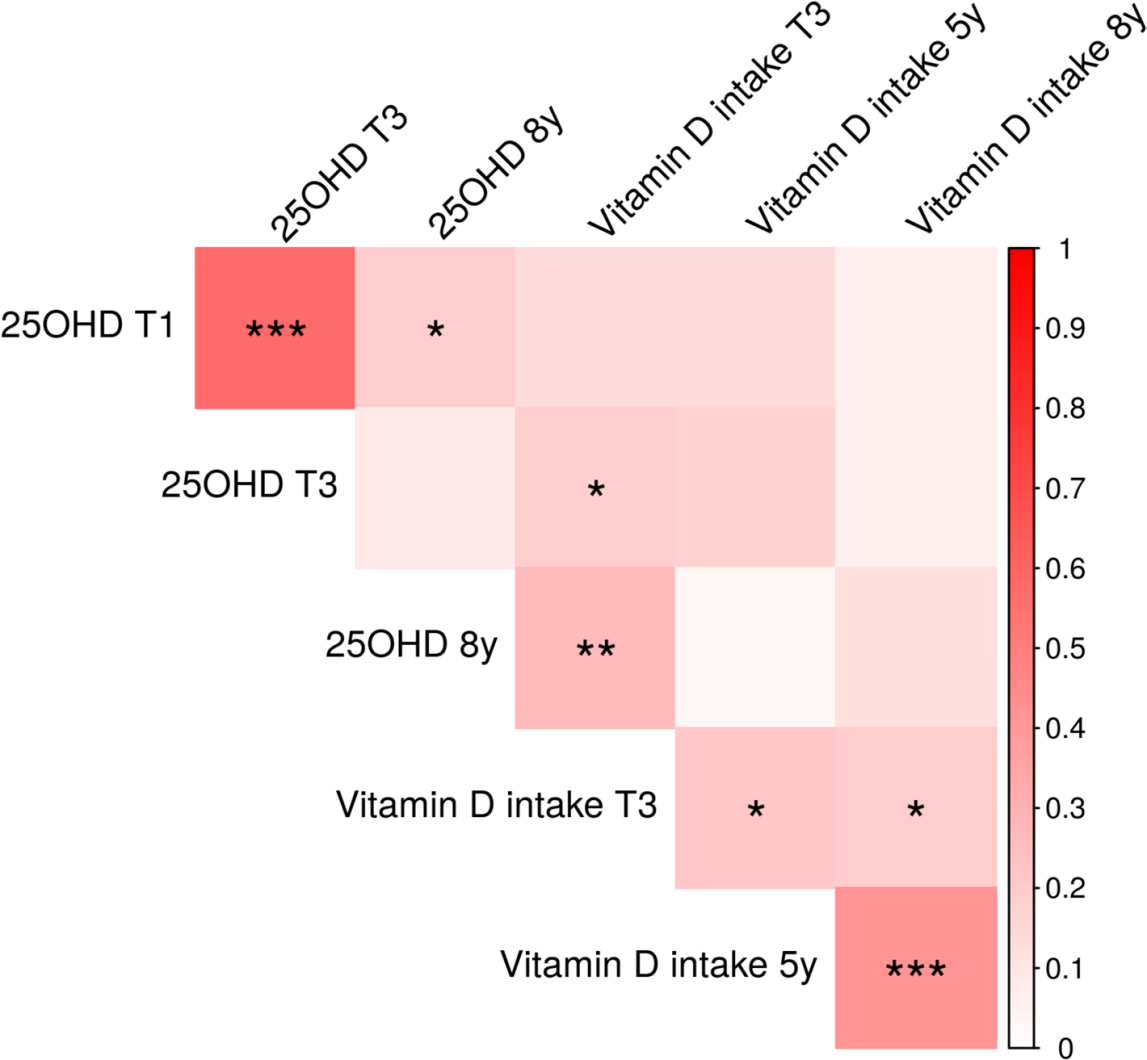
Spearman correlation matrix of season-corrected 25-hydroxyvitamin D (25OHD) concentrations in the first trimester (T1) and third trimester (T3) of pregnancy and in children aged 8 years (8y), and vitamin D intake from vitamin D-rich foods in the third trimester (T3) of pregnancy, in children aged 5 years (5y) and 8 years (8y). The magnitude of the positive correlation is denoted by colours ranging from white to red, with darker colour indicating a stronger correlation. Statistically significant correlations are denoted as follows: *p* < 0.05 = *, *p* < 0.01 = **, and *p* < 0.001 = ***

Season-corrected 25OHD concentrations tracked from the first to the third trimester (rho = 0.58, *p* < 0.001) (Fig. 3). In addition, season-corrected 25OHD from the first trimester tracked with the season-corrected 25OHD in children at the age of 8 years (rho = 0.2, *p* = 0.022). No association was seen between the third trimester 25OHD and 25OHD at 8 years. Reported vitamin D intake in the third trimester was positively correlated with reported vitamin D intake in children aged 5 years (rho = 0.21, *p* = 0.017 and 8 years (rho = 0.19, *p* = 0.04), and with season-corrected 25OHD concentrations in children at age 8 years (rho = 0.26, *p* = 0.002). Reported vitamin D intake at age 5 years was positively correlated with the reported intake at age 8 years (rho = 0.41, *p* < 0.001).

## Discussion

This study showed that the majority of the children at age 8 years had sufficient 25OHD concentrations at 50 nmol/L or above at the time after the expanded vitamin D fortification programme in Sweden, with higher 25OHD concentrations during summer. In addition, vitamin D status and intake during pregnancy appeared to track with levels in childhood, although the correlations were weak.

This is the first study to present data on vitamin D status in early school-aged children since the expansion of the Swedish national vitamin D fortification programme in 2018–2020 [31, 32]. Following the expansion, the children had been exposed to higher vitamin D content and a broader range of fortified foods for approximately 2–5 years prior to blood sampling at early school-age. The mean (SD) crude plasma 25OHD concentration in children in our study (67.8 (15.6) nmol/L) was higher than serum 25OHD concentrations among children aged 10–12 years in the most recent national dietary survey conducted in 2016–17 (52.9 (14.3) nmol/L) [19], prior to the vitamin D fortification expansion. The blood samples in the national survey were collected between March and May, which may partly explain the lower vitamin D levels compared to those observed in our study. Another Swedish study, conducted in the northern part of Sweden on preschool children in 2010–2011, revealed mean serum concentrations of 60 (15) nmol/L in summer and 55 (16) nmol/L in winter, with lower levels among children with darker skin complexions compared to those with fairer skin [13]. Whether the higher 25OHD concentrations observed in our study compared with previous Swedish studies partly reflect the expanded Swedish vitamin D fortification policy should be interpreted with caution. Especially since intake from vitamin D-rich foods was not identified as a significant determinant of 25OHD in our analysis. The homogenous sample of children in GraviD-Child may rather explain these high concentrations. Thus, nationally representative studies are warranted to prospectively evaluate the impact of the expanded vitamin D fortification programme on vitamin D status and intake in school-aged children.

We add to the evidence base that season is a crucial factor to consider when assessing vitamin D status in populations living at higher geographical latitudes [13–17, 20–22, 25, 26]. Based on calculations using data from regions at similar latitudes to our study, UVB availability is insufficient for dermal synthesis of vitamin D for approximately half of the year [11]. We observed that blood sampling in May–October was associated with higher 25OHD concentrations, compared to sampling in November–April. This is in line with previous studies assessing vitamin D status in children in Northern European countries [13, 15, 20–22, 25, 26]. Given the seasonal variation in 25OHD, vitamin D intake from diet and supplements may play a greater role in determining vitamin D status during winter months at northern latitudes. However, due to the relatively small sample size of our study, we were unable to stratify determinants of vitamin D status by season.

The majority of the children in our study had mothers of Northern European origin and a high level of education. Further, the low prevalence of overweight and obesity together with the limited variation in time spent outdoors during summer and in 25OHD concentrations among the children, may help explain the lack of statistically significant determinants that have been identified in other studies as influencing 25OHD levels in children [13, 15, 16, 18–20, 22, 27]. Participants in the follow up at age 8 years were not asked to specify the dosage or brand of supplements they used; therefore, we were unable to calculate their vitamin D intake from supplements. Additionally, we only estimated reported vitamin D intake from vitamin D-rich foods, and not the total intake from all dietary sources. However, the vitamin D questionnaire captured the frequency of intake of fortified dairy products, margarine, and oily fish—foods that have been shown to be important sources in the Swedish national dietary surveys among both children and adults [44, 45].

Tracking between maternal 25OHD concentrations and vitamin D status of their children has been previously demonstrated, although research to date has focused on younger age groups. A Danish mother–child cohort observed associations between maternal 25OHD concentrations during pregnancy and those of their children at 6 months of age, as well as between cord blood 25OHD concentrations and children’s levels at 4 years of age [4]. Moreover, recent research from Norway observed positive correlations between maternal and child 25OHD concentrations measured postnatally at corresponding time points at 6 (r = 0.22) and 12 (r = 0.28) months of age [46]. These correlation coefficients were similar in magnitude to those observed in our mother–child pairs. Tracking of 25OHD concentrations has also been demonstrated throughout childhood and adolescence, with stronger correlations between adjacent time points and higher within-individual correlations than those observed for mother–child pairs. An Icelandic study showed such tracking in young children (r = 0.34) [25], whereas no associations were found in a South African study that investigated correlations from early to late adolescence [47]. The latter study was limited to the small number of participants in the long-term follow up. Unlike the South African study during adolescence, an Australian study demonstrated positive correlations of 25OHD concentrations from early school-age through to 20 years of age (r = 0.40–0.67) [48]. In addition, they identified childhood vitamin D status as a significant predictor of peak bone mass in early adulthood, underscoring the clinical importance of adequate vitamin D levels in early life. We found positive associations between season-corrected 25OHD concentrations in first trimester of pregnancy and in childhood, but not between 25OHD concentrations in the third trimester and the child’s status at age 8. One plausible explanation is that vitamin D status in the first trimester may be predominantly influenced by lifestyle factors, whereas third trimester concentrations may be more affected by physiological changes related to gestational age and hormonal fluctuations, making them less representative of the mother’s typical vitamin D status [35].

The association between maternal vitamin D status in first trimester of pregnancy and that of their children observed in our study is likely influenced by shared habits related to sun exposure and diet. Although we did not have data on vitamin D intake during the first trimester, we found positive correlations between reported intake of vitamin D-rich foods in the third trimester and in children at ages 5 and 8 years. Maternal-child dietary tracking has been demonstrated previously, with similar dietary patterns observed in mothers during pregnancy and in their children at early school-age [49]; however, results for vitamin D intake specifically has not been presented before.

Altogether, our findings, supported by previous longitudinal studies on vitamin D tracking, suggest that vitamin D levels in early life, including the prenatal period, is related with levels later in childhood. Pregnancy may therefore provide a critical window for targeted vitamin D interventions within routine healthcare for those at risk of low vitamin D status. This can have long-term effects on vitamin D intake and status in both mothers and children.

### Strengths and limitations

The main strength of this study is its longitudinal design, with blood samples taken twice during pregnancy, and the long follow up of the children. We also assessed vitamin D intake from vitamin D-rich foods during pregnancy and twice during childhood, and collected information on sun exposure.

Some limitations should be considered in the interpretations of the results. One is the fact that information on vitamin D intake from vitamin D-rich foods, supplement use, and sun exposure was self-reported by mothers during pregnancy and by parents during childhood. Also, the information was collected retrospectively, which may have led to misreporting and introduced recall bias. Nonetheless, our previously validation study indicated that the short vitamin D questionnaire provides valid estimates of vitamin D intake during pregnancy [41], however its validity among children is uncertain.

Another limitation is selection bias. The GraviD study was a population-representative sample of the Swedish pregnant population at the time of data collection [35]. However, the relatively small and homogeneous sample that consented to participate in the clinical follow up of children may have introduced selection bias, and thereby limiting the external validity of the results. Compared to the whole GraviD study (*n* = 2125), the mother–child pair included in this study (*n* = 145) had a slightly older maternal age, a lower pre-pregnancy BMI, a higher proportion with a university education level and of northern European origin. However, despite the small sample size, limited variation in some variables and reduced statistical power, we find that vitamin D status and intake may track from pregnancy to childhood, a finding that may be valid despite selection bias.

Lastly, some uncertainties related to the assessment of 25OHD need to be acknowledged. A major limitation is the use of different analytical methods for 25OHD. Maternal samples during pregnancy were analysed using LC–MS/MS, whereas children’s samples were analysed using CMIA. However, tracking of vitamin D status in mother–child pairs was assessed using Spearman correlation, which is based on rank order rather than absolute 25OHD concentrations and is therefore less sensitive to systematic differences between methods. Best practice would have been to retrospectively standardize 25OHD concentrations according to the Vitamin D Standardization Program (VDSP). This involves however remeasuring a statistically defined subsample of stored samples across the 25OHD concentration range and subsequently developing a calibration equation to adjust previously measured values [50]. However, in our study, stored samples were not available for the children’s samples, precluding such standardization. Since 25OHD concentrations were not standardized according to VDSP, absolute concentrations and the estimated prevalences of vitamin D insufficiency and deficiency in children should be interpreted with caution.

Another limitation is that concentrations of 25OHD were analysed in serum in pregnancy and in plasma at clinical follow up in children. Differences between plasma and serum 25OHD concentrations analysed by LC–MS/MS have been reported in pregnancy, with plasma tending to be higher than serum [51]. However, others have found similar concentrations between the two matrices in a non-pregnant population and in samples from the umbilical cord [52, 53]. It is therefore unclear whether, and to what extent, differences between matrices affect 25OHD concentrations. But again, as our analyses are based on ranks, the findings are less sensitive to such systematic differences.

## Conclusion

Among early school-aged children in Sweden with generally adequate vitamin D levels, season was the only significant factor for determining vitamin D status. Maternal vitamin D status and intake were associated with childhood levels, and although the correlations were weak, our findings suggest tracking from pregnancy to childhood, highlighting pregnancy as a potential period for targeted vitamin D interventions that could have long-term effects on children’s levels.

## Supplementary Information

Supplementary Material 1.

## Data Availability

Data from the GraviD study cannot be made freely available since the consent given by the participants does not open for storage of data on an individual level in repositories or journals, and as the data are subject to secrecy in accordance with the Swedish Public Access to Information and Secrecy Act [Offentlighets-och sekretesslagen, OSL, 2009:400], but can be made available to researchers upon request. Access to data requires approval from the Swedish Ethical Review Authority.
