# Supplementary material for: Vitamin D status and intake in early school-aged children is tracking from pregnancy—the Swedish GraviD-Child study
Source: BMC Pediatr. 2026 Apr 27;26:384. doi: 10.1186/s12887-026-06914-3 (PMC13123185; doi:10.1186/s12887-026-06914-3)
Supplement: Supplementary file 1 — Supplementary Material 1. [file 12887_2026_6914_MOESM1_ESM.pdf]

Supplementary Information

Vitamin D status and intake in early school-aged children is tracking from pregnancy - the Swedish GraviD-Child study

BMC Pediatrics

Mathilda Forsby, Linnea Bärebring, Anna Amberntsson, Frida Dangardt, Anna Winkvist, Hanna Augustin

Corresponding Author

Mathilda Forsby

mathilda.forsby@gu.se

Department of Internal Medicine and Clinical Nutrition, Institute of Medicine, University of Gothenburg, Gothenburg, Sweden.

Supplementary Information on Methods

Questions used in the GraviD-Child follow up at 8 years of age

1. **On a regular weekday outside school hours, please estimate how many hours per day the child usually spends outdoors.**

   Summer ………… hours per day

   Winter ………… hours per day

2. **Has the child travelled abroad during the past 6 months? ☐ NO ☐ YES**
   If YES: To which destination(s)? ....
   For how long? ....
   When did the child return home from the trip? ....

3. **How does the child’s skin react during the first hour in the sun in spring?**
   ☐ Always burns, never tans (minimal pigmentation)
   ☐ Always burns, tans with difficulty (slow pigmentation)
   ☐ Sometimes mild burn, tans about average (light pigmentation, skin becomes light brown)
   ☐ Rarely burns, tans easily (quick pigmentation, skin becomes brown)
   ☐ Minimally burns, tans very easily (strong pigmentation, skin becomes dark brown)
   ☐ Never burns, tans very easily (strongly pigmented, skin is black)

4. **What is the child’s eye colour (choose only one option)?**
   ☐ Hazel brown
   ☐ Dark brown
   ☐ Blue
   ☐ Green

5. **Does your child take any of the following dietary supplements?**

	Frequency per week					Dosage per intake
**Supplement**	**6-7**	**4-5**	**1-3**	**<1**	**0**	**(1 / 2 / 3+)**
Fish liver oil	☐	☐	☐	☐	☐	☐ ☐ ☐
Multivitamin	☐	☐	☐	☐	☐	☐ ☐ ☐
Omega-3	☐	☐	☐	☐	☐	☐ ☐ ☐
Other (brand/type): ………………	☐	☐	☐	☐	☐	☐ ☐ ☐

<u>Please answer how the child has eaten during the past 2 months</u>

6. **Does the child eat oily fish? ☐ NO ☐ YES**
   If YES, how often?
   ☐ At least 2 times/week
   ☐ 3-4 times/month
   ☐ 1-2 times/month
   ☐ Less often

7. **Does the child drink milk (including oat or soy drink), or is milk part of the child's diet?**
   ☐ NO ☐ YES
   If YES, how much milk does the child consume per day?
   ☐ More than 3 dl
   ☐ 1-3 dl
   ☐ Less than 1 dl

8. **Is fermented milk (e.g. yoghurt and sour milk, including oat or soy yoghurt) part of the child's diet? ☐ NO ☐ YES**
   If YES, how much fermented milk does the child consume per day?
   ☐ More than 3 dl
   ☐ 1-3 dl
   ☐ Less than 1 dl

9. **Does the child use margarine as spread on sandwiches (e.g., Bregott, Lätta, Flora, Milda, Becel)? ☐ NO ☐ YES**
   If YES, how many sandwiches with margarine does the child eat?
   ☐ At least 4/day
   ☐ 2-3/day
   ☐ 4-7/week
   ☐ 1-3/week
   ☐ Fewer

Supplementary Figures

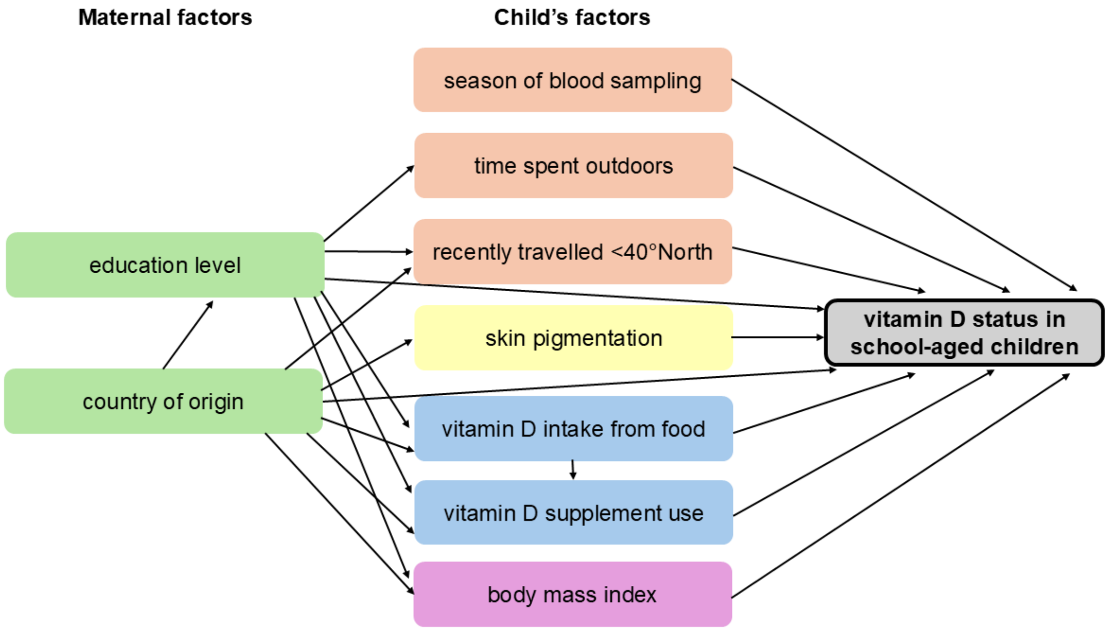

Figure S1. Potential determinants of vitamin D status in school-aged children visualised by a directed acyclic graph. Colours represent the child's sun exposure (orange), child's skin pigmentation (yellow), child's vitamin D intake (in blue), child's body mass index (purple), and maternal demographic factors (in green).

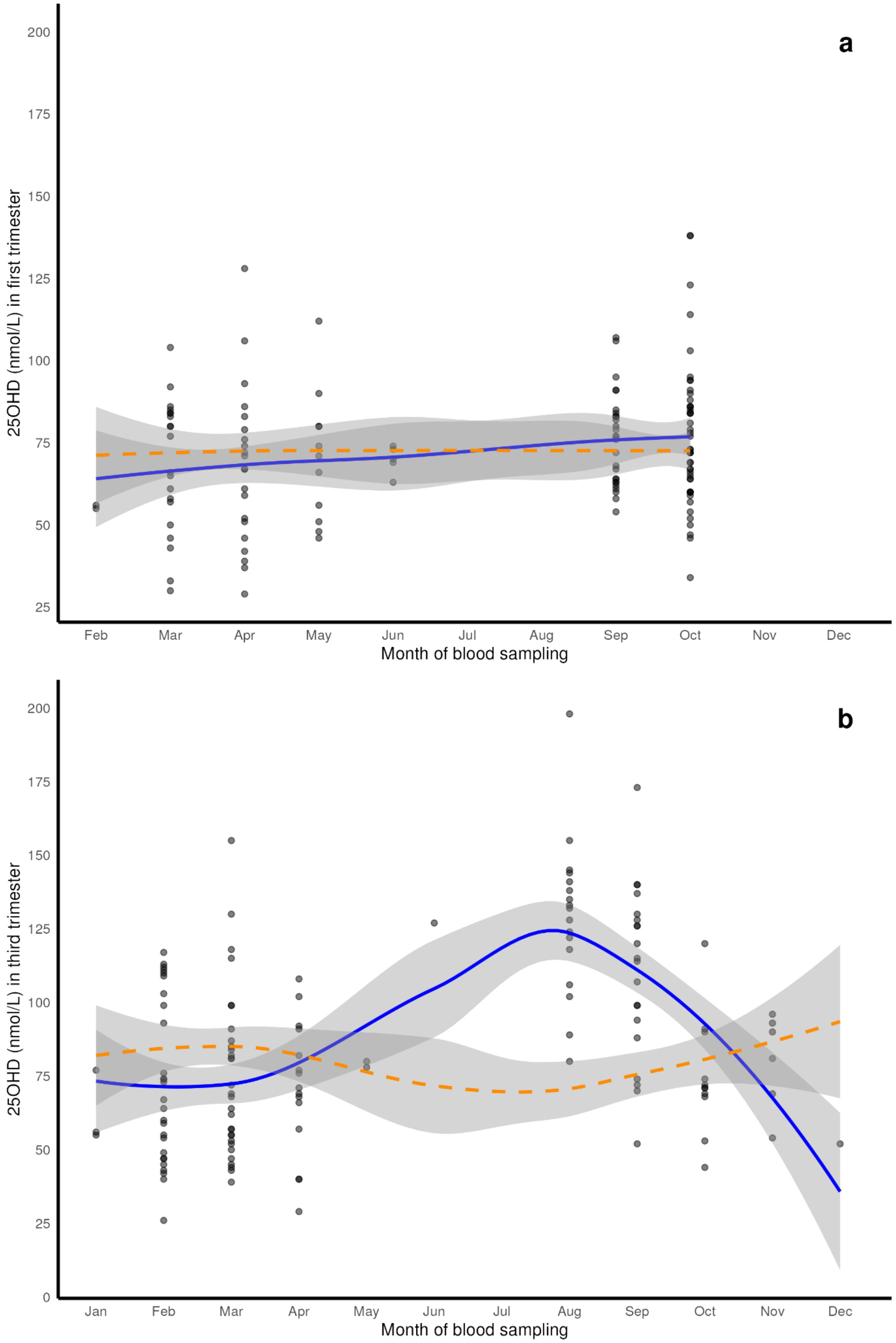
a
25OHD (nmol/L) in first trimester
200
175
150
125
100
75
50
25
Feb
Mar
Apr
May
Jun
Jul
Aug
Sep
Oct
Nov
Dec
Month of blood sampling
b
25OHD (nmol/L) in third trimester
200
175
150
125
100
75
50
25
0
Jan
Feb
Mar
Apr
May
Jun
Jul
Aug
Sep
Oct
Nov
Dec
Month of blood sampling

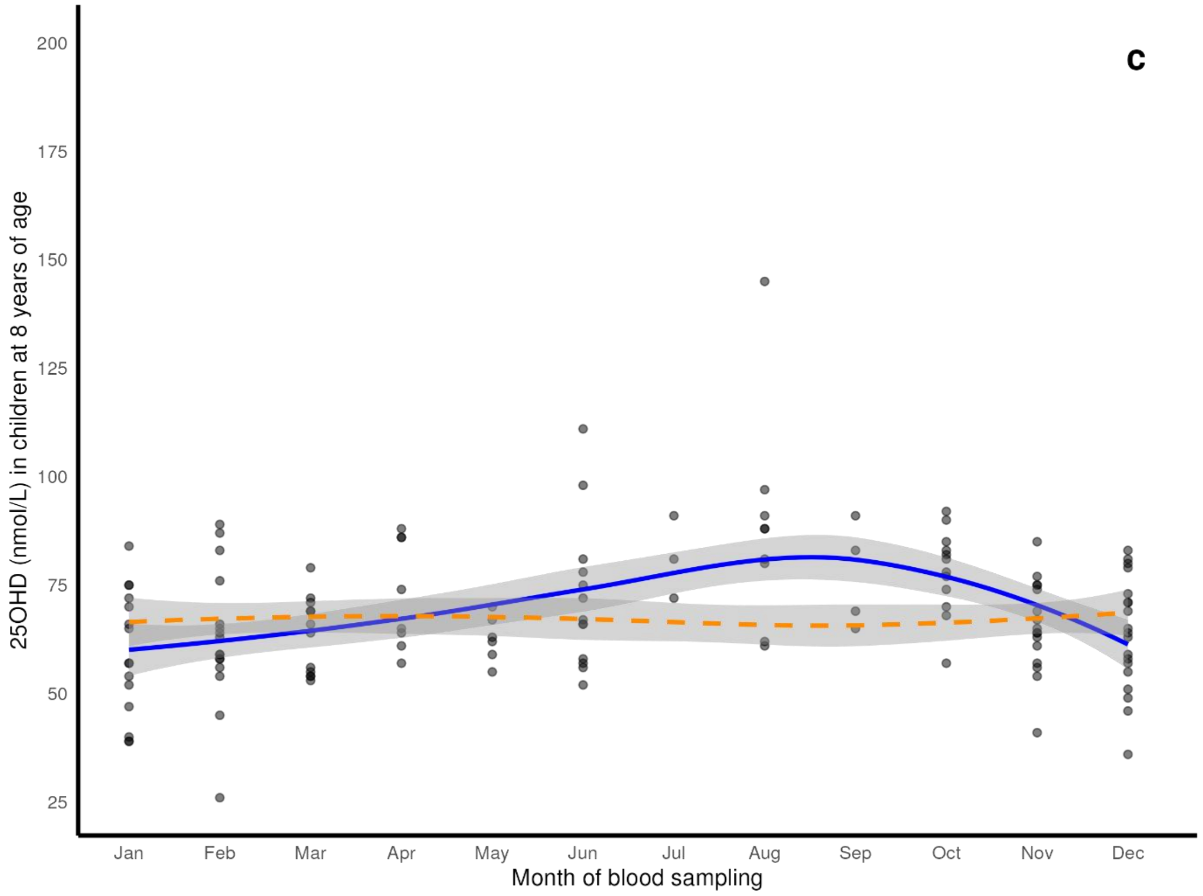

Figure S2. Scatterplots of the seasonal variation in 25-hydroxyvitamin D (25OHD) concentrations in mothers during the first (a) and third (b) trimesters of pregnancy, and in children at 8 years of age (c) (N=133). Loess smoothed curves of crude 25OHD concentration (solid, blue line) and the season-corrected 25OHD concentration (dotted, orange line) with a 95% confidence interval (grey area) are plotted.
